# Block Copolymer Nanoparticles are Effective Dispersants for Micrometer-Sized Organic Crystalline Particles

**DOI:** 10.1021/acsami.1c08261

**Published:** 2021-06-21

**Authors:** Derek H. H. Chan, Emily L. Kynaston, Christopher Lindsay, Philip Taylor, Steven P. Armes

**Affiliations:** † Dainton Building, Department of Chemistry, University of Sheffield, Brook Hill, Sheffield, South Yorkshire S3 7HF, U.K.; ‡ Syngenta, Jealott’s Hill International Research Centre, Bracknell, Berkshire RG42 6EY, U.K.

**Keywords:** azoxystrobin, nanoparticles, microparticles, suspension concentrates, RAFT polymerization, block copolymer, polymerization-induced self-assembly

## Abstract

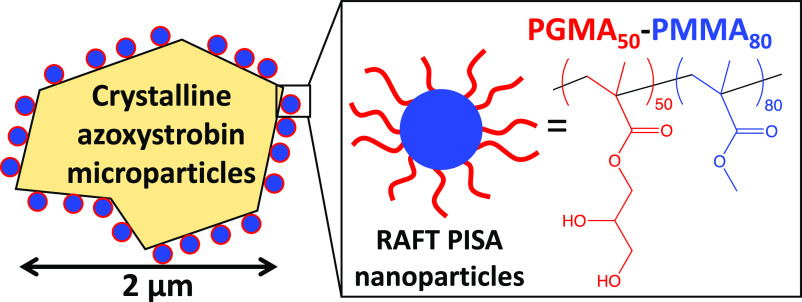

Well-defined sterically stabilized diblock copolymer nanoparticles of 29 nm diameter are prepared by RAFT aqueous emulsion polymerization of methyl methacrylate using a dithiobenzoate-capped poly(glycerol monomethacrylate) precursor. These nanoparticles are evaluated as a dispersant for the preparation of organic crystalline microparticles via ball milling. This is exemplified for azoxystrobin, which is a broad-spectrum fungicide that is widely used to protect various food crops. Laser diffraction and optical microscopy studies indicate the formation of azoxystrobin microparticles of approximately 2 μm diameter after ball milling for 10 min at 400 rpm. Nanoparticle adsorption at the surface of these azoxystrobin microparticles is confirmed by electron microscopy studies. The extent of nanoparticle adsorption on the azoxystrobin microparticles can be quantified using a supernatant assay based on solution densitometry. This technique indicates an adsorbed amount of approximately 5.5 mg m^–2^, which is sufficient to significantly reduce the negative zeta potential exhibited by azoxystrobin. Moreover, this adsorbed amount appears to be essentially independent of the nature of the core-forming block, with similar data being obtained for both poly(methyl methacrylate)- and poly(2,2,2-trifluoroethyl methacrylate)-based nanoparticles. Finally, X-ray photoelectron spectroscopy studies confirm attenuation of the underlying N1s signal arising from the azoxystrobin microparticles by the former adsorbed nanoparticles, suggesting a fractional surface coverage of approximately 0.24. This value is consistent with a theoretical surface coverage of 0.25 calculated from the adsorption isotherm data. Overall, this study suggests that sterically stabilized diblock copolymer nanoparticles may offer a useful alternative approach to traditional soluble copolymer dispersants for the preparation of suspension concentrates affecting the context of agrochemical applications.

## Introduction

**Figure 1:**
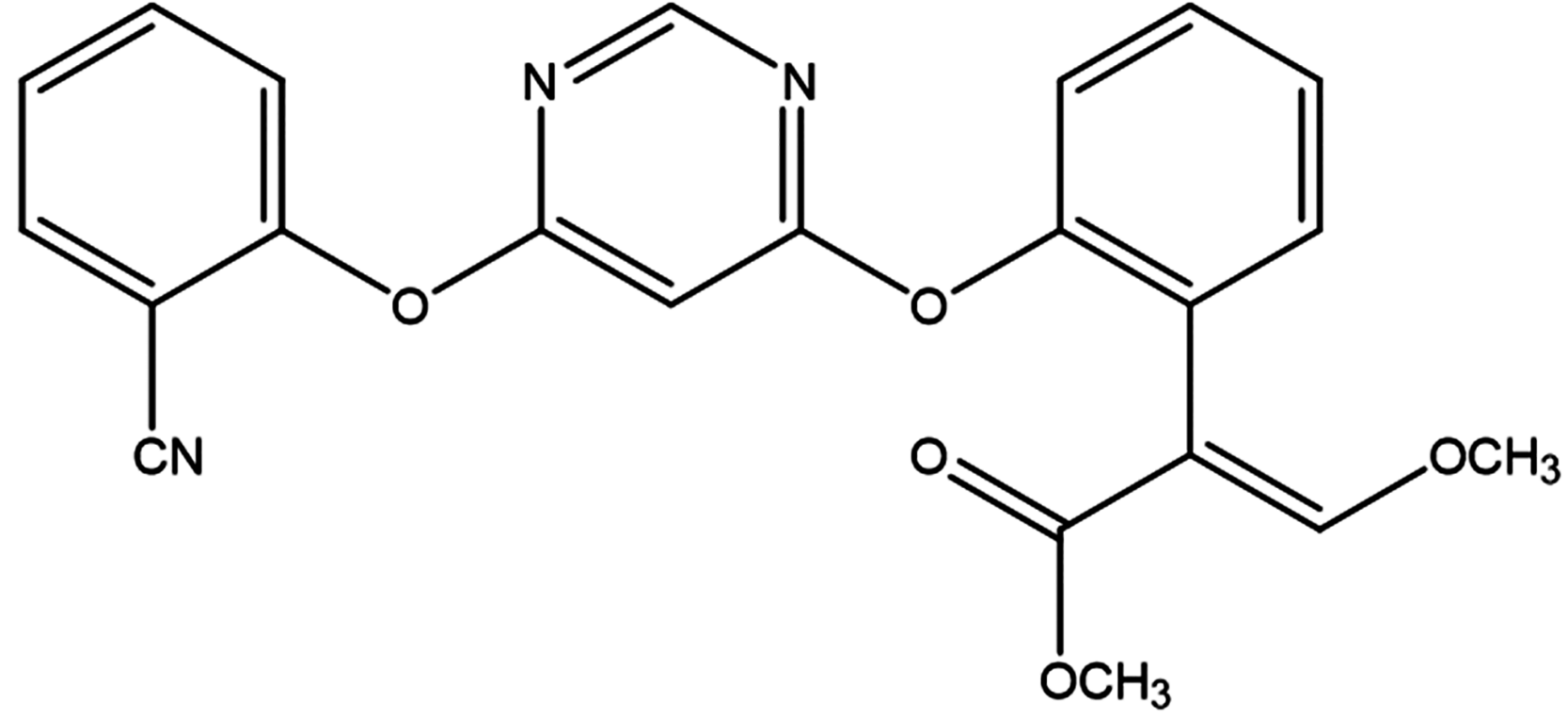
Chemical structure of azoxystrobin, a member of the strobilurin family. This broad-spectrum fungicide is used to prevent a wide range of crop diseases.

Azoxystrobin is a broad-spectrum strobilurin fungicide that is widely used for the control of a range of diseases in cereals, brassicae, beans, asparagus, peas, oil seed rape, potatoes, carrots, alliums, strawberries, lettuce, and other food crops.^1,2^ This molecule preferentially binds at the quinol outer binding site of the cytochrome b-c1 complex relative to ubiquinone (coenzyme Q10), which transports electrons to this protein. This prevents ATP production and hence inhibits mitochondrial respiration.^3^ The chemical structure of azoxystrobin is shown in Figure 1. It is an organic crystalline compound with a melting point of 116 °C, and it has a relatively low aqueous solubility of 6.7 mg dm^–3^. Consequently, azoxystrobin is usually formulated as a concentrated aqueous dispersion of micron-sized particles (also known as “suspension concentrates” or SCs) using various water-soluble synthetic polymers or biopolymers as dispersants.^4^ Recently, submicrometer-sized azoxystrobin particles have been prepared and shown to exhibit greater efficacy.^5,6^ Such colloidal dispersions were reported to be “self-dispersible” but in fact a commercial *Pluronic*-type block copolymer was used for their preparation.^5^

In the colloid science literature, there are many examples of the physical adsorption of small particles onto large particles. Often, such studies involve model systems,^7−12^ but potential applications include new routes for (i) core–shell particles for paints and coating applications^13,14^ and (ii) polymer–silica nanocomposite particles.^15−17^ In addition, we recently reported that 30 nm-diameter diblock copolymer nanoparticles can act as a particulate dispersant for 470 nm-diameter silica particles, which serve as a model pigment.^18^ At pH 7, the nanoparticles acquired cationic character and their electrostatic adsorption onto the anionic silica particles led to a fractional surface coverage of 0.42.

Over the past decade or so, polymerization-induced self-assembly (PISA) has become widely recognized as a powerful and versatile platform technology for the rational synthesis of sterically stabilized diblock copolymer nanoparticles of controllable size and shape.^19−26^ In essence, PISA involves using a soluble precursor block to grow an insoluble block in a suitable solvent, with *in situ* micellar self-assembly occurring at a certain critical degree of polymerization (DP). Of particular relevance to the present study, PISA can be conducted in aqueous media using reversible addition–fragmentation chain transfer (RAFT) polymerization.^26−28^ Depending on the aqueous solubility of the vinyl monomer, this may involve either an aqueous emulsion or an aqueous dispersion formulation.^29−31^ A wide range of water-soluble stabilizer blocks have been employed, including non-ionic, anionic, cationic, and zwitterionic examples.^32−36^ Similarly, various water-insoluble core-forming blocks have been examined, including polystyrene, poly(methyl methacrylate), poly(*n*-butyl acrylate), and poly(benzyl methacrylate).^32,37−40^ In many cases, the sole copolymer morphology is kinetically trapped spheres, regardless of the diblock composition that is targeted.^37,41−44^ Such nanoparticles have been evaluated for coating applications^39^ and also as Pickering emulsifiers for the preparation of oil-in-water emulsions.^45^

**Scheme 1:**
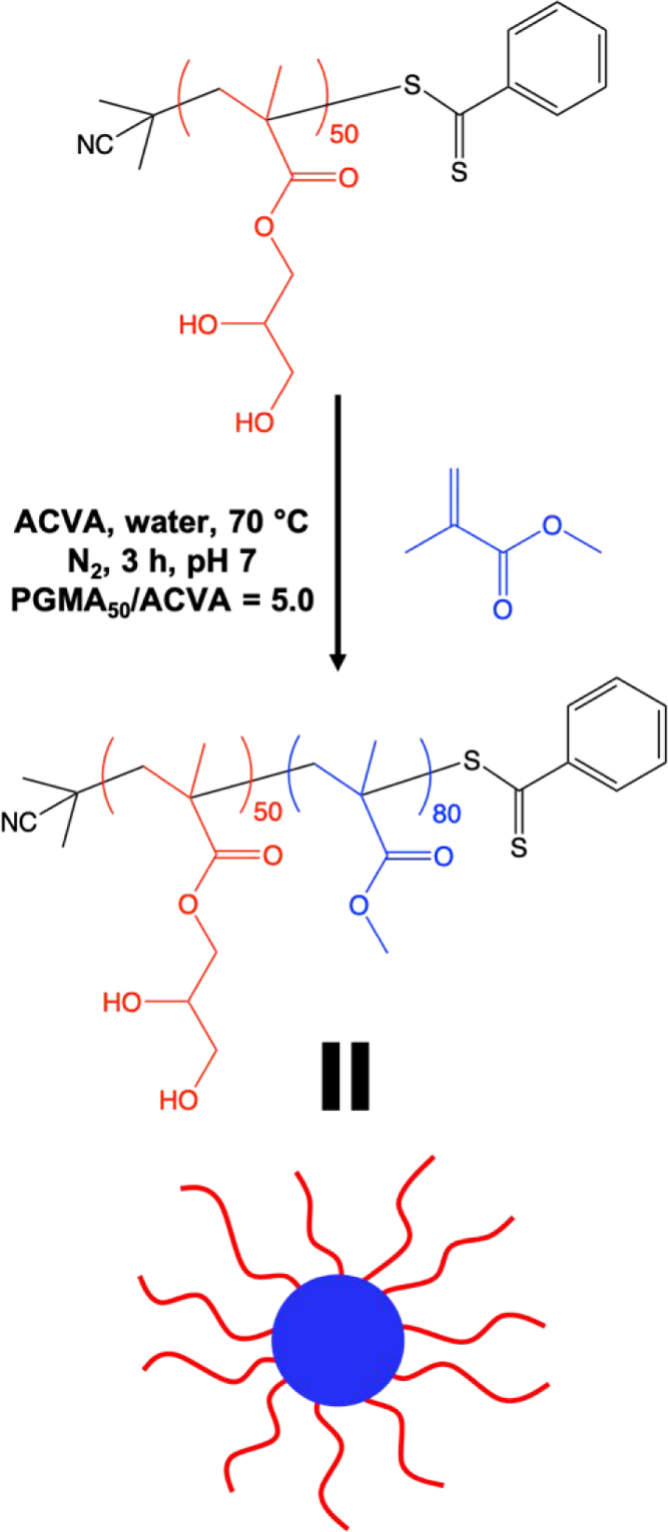
Synthesis of PGMA_50_-PMMA_80_ Diblock Copolymer Nanoparticles by RAFT Aqueous Emulsion Polymerization of MMA Using a Water-Soluble Poly(glycerol monomethacrylate) (PGMA_50_) Precursor at 70 °C

**Scheme 2:**
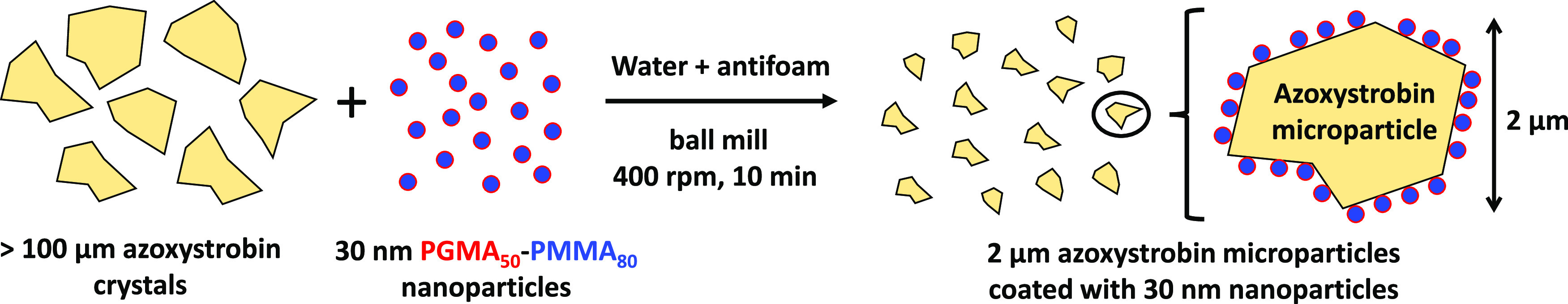
Schematic Representation of the Preparation of 2 μm Azoxystrobin Microparticles in the Form of a 20% w/w Aqueous Suspension Concentrate by Ball Milling Macroscopic Azoxystrobin Crystals in the Presence of an Aqueous Dispersion of 30 nm-Diameter PGMA_50_-PMMA_80_ Nanoparticles [N.B. Components Are Not Drawn to Scale]

Herein, we chain-extend a water-soluble poly(glycerol monomethacrylate) (PGMA) precursor via RAFT aqueous emulsion polymerization of methyl methacrylate (MMA) to prepare diblock copolymer spheres (see Scheme 1). We demonstrate that such sterically stabilized nanoparticles are effective dispersants for organic crystalline microparticles, enabling the production of aqueous suspension concentrates (SCs) via ball milling. This finding is exemplified for azoxystrobin, one of the world’s most widely used fungicides (see Scheme 2). To aid characterization of such azoxystrobin microparticles, we also prepared nanoparticles of comparable size prepared via RAFT aqueous emulsion polymerization of 2,2,2-trifluoroethyl methacrylate (TFEMA) using the same PGMA steric stabilizer.^46^ This is because this semi-fluorinated monomer offers superior electron contrast when characterizing the nanoparticle-coated microparticles by transmission electron microscopy.

## Experimental Section

### Materials

MMA (99%), TFEMA (99%), 4,4′-azobis(4-cyanopentanoic acid) (ACVA; 98%), 2-cyano-2-propyl benzodithioate (CPDB; 97%), and Triton X-100 were purchased from Sigma-Aldrich (UK). Glycerol monomethacrylate (GMA) was donated by GEO Specialty Chemicals (Hythe, UK), and the commercial dispersant Morwet D-425 was obtained from AkzoNobel (Sweden). Azoxystrobin was provided by Syngenta (Jealott’s Hill, UK). The antifoaming agent silicone SAG1572 was purchased from Momentive (Germany), and 1.0 mm zirconium aluminum oxide beads were purchased from Sigmund-Lindner (Germany). Deionized water was used for all experiments.

### Synthesis Protocols

#### Synthesis of the PGMA_50_ Precursor by RAFT Aqueous Solution Polymerization

The GMA monomer (30.0 g, 187 mmol), CPDB (0.589 g, 2.66 mmol; target PGMA DP = 70), ACVA initiator (0.149 g, 0.53 mmol; CPDB/ACVA molar ratio = 5.0), and ethanol (46.5 g, 60% w/w) were weighed into a 250 mL round-bottom flask. The flask was then immersed in an ice bath, and the solution was deoxygenated using a stream of N_2_ gas for 30 min. The reaction mixture was then placed in an oil bath set at 70 °C for 165 min and a final GMA conversion of 71% was determined by ^1^H NMR spectroscopy. The solution was diluted with methanol (30 mL), and the crude polymer was then precipitated into a ten-fold excess of dichloromethane to remove the unreacted monomer and other impurities. This precursor was redissolved in methanol and precipitated twice before using ^1^H NMR spectroscopy to determine a mean DP of 50 via end-group analysis (the integrated aromatic signal at 7.4–7.8 ppm was compared to that of the methacrylic backbone at 0.7–2.5 ppm).

#### Synthesis of PGMA_50_-PMMA_80_ Diblock Copolymer Nanoparticles by RAFT Aqueous Emulsion Polymerization

An aqueous emulsion comprising the PGMA_50_ precursor (0.150 g, 18.2 μmol), MMA monomer (0.146 g, 1.46 mmol), ACVA initiator (1.0 mg, 3.65 μmol, CTA/ACVA molar ratio = 5.0), and deionized water (2.675 g, 10% w/w solution) was made up in a 20 mL round-bottom flask. This flask was immersed in an ice bath and the emulsion was deoxygenated using a stream of N_2_ gas for 30 min. The flask was then placed in an oil bath set at 70 °C, and the ensuing polymerization was quenched after 3 h by exposing the flask contents to air while cooling to 20 °C.

#### Synthesis of PGMA_50_-PTFEMA_80_ Diblock Copolymer Nanoparticles by RAFT Aqueous Emulsion Polymerization

An aqueous emulsion comprising the PGMA_50_ precursor (0.150 g, 18.2 μmol), TFEMA monomer (0.245 g, 1.46 mmol), ACVA initiator (1.0 mg, 3.65 μmol, CTA/ACVA molar ratio = 5.0), and deionized water (3.568 g, 10% w/w solution) was made up in a 20 mL round-bottom flask. This flask was immersed in an ice bath, and the emulsion was deoxygenated using a stream of N_2_ gas for 30 min. The flask was then placed in an oil bath set at 70 °C, and the ensuing polymerization was quenched after 6 h by exposing the flask contents to air while cooling to 20 °C.

#### Preparation of SCs by Ball Milling

Azoxystrobin (3.00 g), PGMA_50_-PMMA_80_ nanoparticles (0.375 g, 2.5% w/w), SAG1572 antifoaming agent (0.15 g, 1.0% w/w), and deionized water (11.48 g, 76.5%) were added to a 50 mL Retsch zirconium oxide-coated jar along with 1.0 mm ceramic beads (15.0 g). A Retsch PM 100 planetary ball mill was used to mill this suspension at 400 rpm for 10 min. The beads were removed by filtration to afford a 20% w/w suspension concentrate.

#### Centrifugal Purification of SCs

SCs were centrifuged for 5 min at 5000 rpm using a Thermo Heraeus Biofuge Pico centrifuge and the aqueous supernatant containing excess copolymer nanoparticles was carefully decanted. The sedimented microparticles were redispersed using deionized water. Two further centrifugation/redispersion cycles were performed prior to characterization of the purified nanoparticle-coated azoxystrobin microparticles.

#### Examination of the Stability of SCs Using a Surfactant Challenge

The suspension concentrates (1.0 g) and Triton X-100 surfactant (10.0 mg, 1.0% w/w) were weighed into a 5 mL vial, which was placed on a roller mixer for 24 h at 20 °C prior to transmission electron microscopy (TEM) analysis.

### Characterization Techniques

#### Dynamic Light Scattering and Aqueous Electrophoresis

A Malvern Zetasizer NanoZS instrument was used to perform both DLS and aqueous electrophoresis studies with an aqueous dispersion concentration of 0.50% w/w being used in each case. Hydrodynamic *z*-average diameters were determined at 20 °C using a scattering angle of 173°, and measurements were averaged over three runs. Aqueous electrophoresis experiments utilized 1 mM KCl as background salt, and the solution pH being adjusted as required with either HCl or NaOH. The Smoluchkowski approximation was used to calculate zeta potentials (also averaged over three measurements) via the Henry equation.

#### Gel Permeation Chromatography

Molecular weight distributions were assessed for the PGMA_50_ precursor, the PGMA_50_-PMMA_80_ diblock copolymer by GPC analysis, and the PGMA_50_-PTFEMA_80_ diblock copolymer at 60 °C using DMF eluent (containing 10 mM LiBr), two Agilent PL gel 5 μm Mixed-C columns connected to a Varian 290-LC pump injection module, and a Varian 390-LC multidetector suite (refractive index detector). A series of near-monodisperse poly(methyl methacrylate) standards ranging from *M*_n_ = 645 g mol^–1^ to 618 000 g mol^–1^ were used for calibration at a flow rate of 1.0 mL min^–1^.

#### Optical Microscopy

A Cole-Palmer optical microscope fitted with a Moticam camera and an LCD tablet was used for imaging both the original coarse azoxystrobin crystals and the much finer azoxystrobin microparticles obtained after milling.

#### Transmission Electron Microscopy

Copper/palladium TEM grids (Agar Scientific, UK) were coated with a thin film of amorphous carbon and then treated with a plasma glow discharge for 30 s. A 10 μL droplet of a 0.10% w/aqueous dispersion (or SC) was placed on each grid for 60 s before blotting. Each particle-loaded grid was stained for 20 s using uranyl formate (9.0 μL of 0.75% w/w solution) before removing excess stain and drying under vacuum. TEM studies were performed at 100 kV using a Philips CM100 instrument equipped with a Gatan 1 k CCD camera.

#### Laser Diffraction

The initial coarse azoxystrobin crystals and the milled azoxystrobin microparticles were sized by laser diffraction using a Malvern Mastersizer 3000 instrument equipped with a Hydro EV wet dispersion unit set at 2000 rpm. The volume-average particle diameter, *d*(0.5), was calculated by averaging over five measurements and assuming an absorption index of 0.10.

#### Scanning Electron Microscopy

Scanning electron microscopy (SEM) images were recorded using an FEI Inspect-F instrument at an accelerating voltage of 10 kV. Samples were allowed to dry overnight on thin glass slides and then sputter-coated with a thin overlayer of gold before imaging.

#### Solution Densitometry

An Anton Paar DMA 4500 M density meter was used to determine the solution densities of 0.50–5.00% w/w aqueous dispersions of PGMA_50_-PMMA_80_ nanoparticles and also various aqueous supernatants obtained after centrifugation of a series of SCs at 20 °C.

#### X-ray Photoelectron Spectroscopy

Azoxystrobin, PGMA_50_-PTFEMA_80_ nanoparticles, PGMA_50_-PMMA_80_ nanoparticles, and the two types of nanoparticle-coated azoxystrobin microparticles were placed in turn on indium foil and analyzed using a Kratos Axis Supra X-ray photoelectron spectrometer. Survey spectra were recorded for each sample using a step size of 0.50 eV. High resolution core-line spectra were recorded for each element of interest using a step size of 0.05 eV.

## Results and Discussion

A PGMA_50_ precursor was synthesized by RAFT solution polymerization of GMA in methanol using a dithiobenzoate-based RAFT agent (CPDB). After purification, ^1^H NMR spectroscopy was used to calculate a mean DP of 50 for this homopolymer by end-group analysis. This precursor was then chain-extended via RAFT aqueous emulsion polymerization of MMA to afford PGMA_50_-PMMA_80_ nanoparticles, with essentially full conversion being achieved within 3 h at 70 °C (Scheme 1).

**Figure 2:**
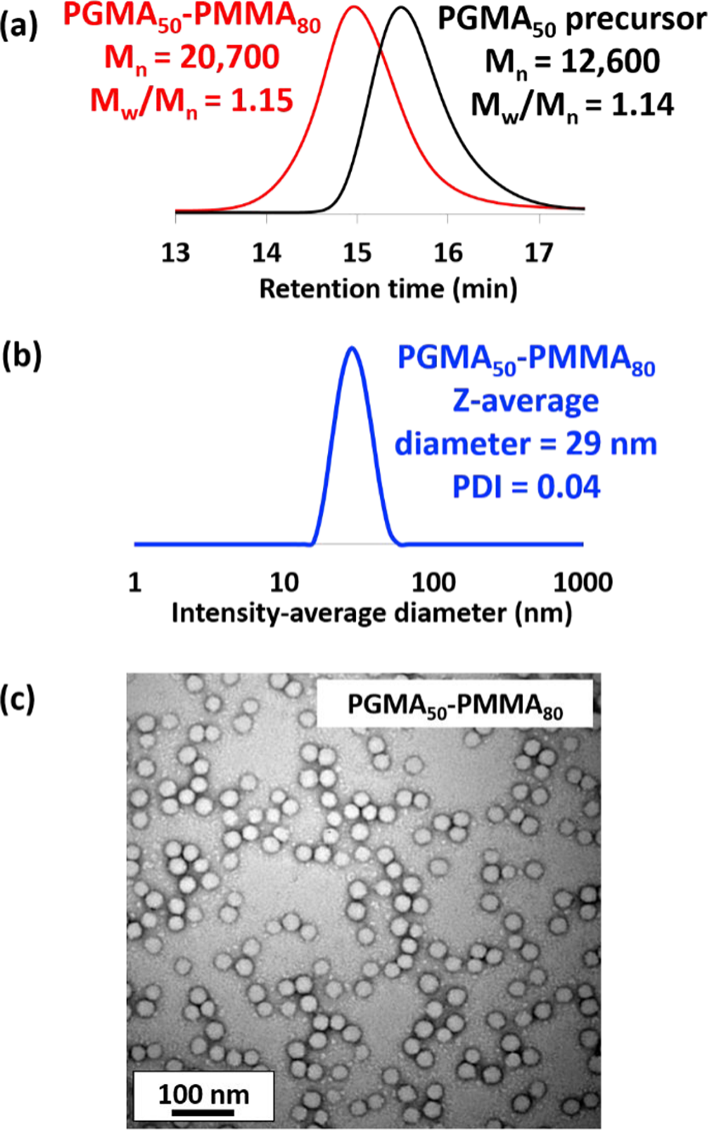
(a) GPC curves recorded for the PGMA_50_ precursor and PGMA_50_-PMMA_80_ nanoparticles; (b) DLS intensity-average particle size distribution (plus *z*-average diameter and polydispersity, PDI); and (c) TEM image recorded for PGMA_50_-PMMA_80_ spherical nanoparticles.

DMF GPC analysis (Figure 2a) confirmed the expected increase in molecular weight for the PGMA_50_-PMMA_80_ diblock copolymer chains relative to the PGMA_50_ homopolymer precursor, and the relatively low dispersity (*M*_w_/*M*_n_ = 1.15) is consistent with a well-controlled RAFT polymerization. The PGMA_50_-PMMA_80_ diblock copolymer nanoparticles were characterized in terms of their particle size using DLS and TEM (see Figure 2b,c). DLS studies indicated a *z*-average diameter of 29 ± 4 nm, while TEM analysis confirmed a spherical morphology and a number-average diameter of 25 ± 3 nm. The same protocol was also used to prepare the equivalent PGMA_50_-PTFEMA_80_ nanoparticles of comparable size (see Scheme S1 and Figure S1).

**Figure 3:**
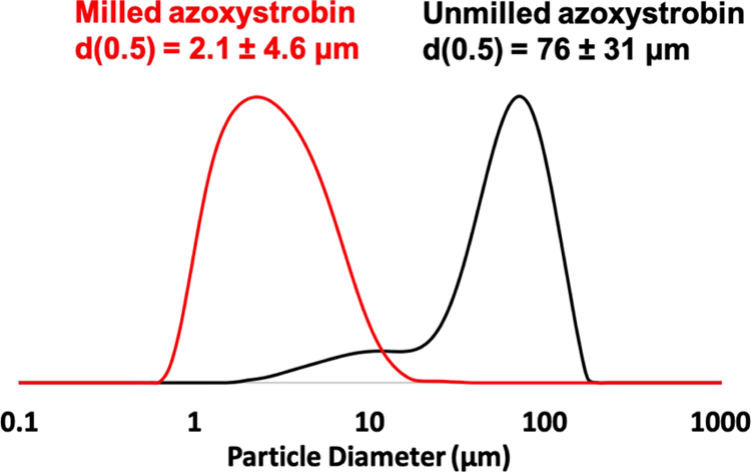
Laser diffraction particle size distribution curves (based on a volume-weighted average) recorded for the original coarse azoxystrobin particles and the much finer PGMA_50_-PMMA_80_ nanoparticle-coated azoxystrobin microparticles obtained after ball milling.

**Figure 4:**
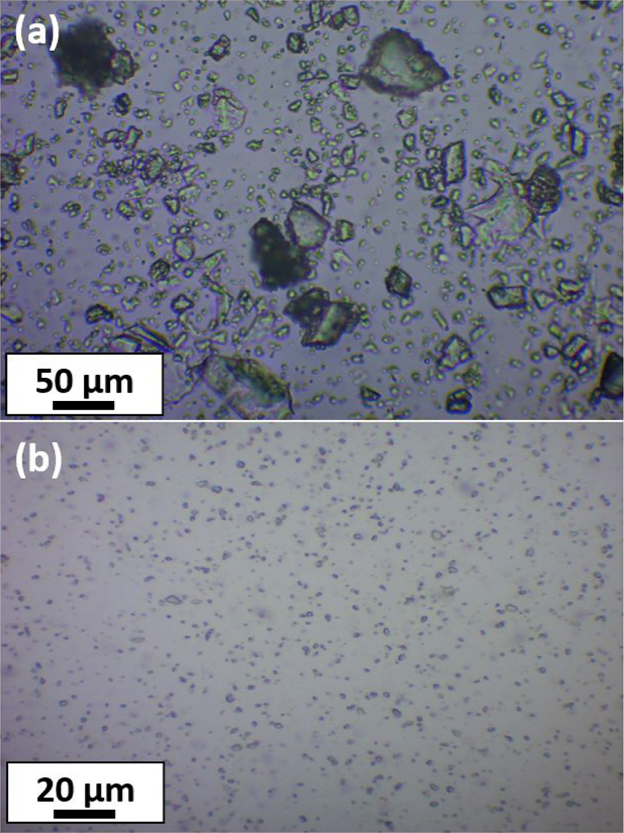
Optical microscopy images of (a) unmilled azoxystrobin crystals and (b) azoxystrobin microparticles after ball milling in the presence of PGMA_50_-PMMA_80_ nanoparticles.

In the context of agrochemical science, hydrophobic organic crystalline compounds are typically milled in the presence of a suitable dispersant to prepare suspension concentrate formulations.^47,48^ Accordingly, ball milling of azoxystrobin crystals was performed in the presence of an aqueous dispersion of PGMA_50_-PMMA_80_ nanoparticles, which was used instead of a conventional water-soluble copolymer dispersant (Scheme 2). The size distributions obtained by laser diffraction for the initial azoxystrobin crystals and the final azoxystrobin microparticles after milling in the presence of such nanoparticles are shown in Figure 3. A substantial reduction in the volume-average particle diameter from 76 μm to approximately 2 μm was achieved after milling for just 10 min under the stated conditions. These laser diffraction data were supported by optical microscopy studies, which also indicated a marked reduction in the mean size of the azoxystrobin crystals (Figure 4a,b). Clearly, the PGMA_50_-PMMA_80_ nanoparticles can act as both a wetting agent and an effective dispersant, which enables a free-flowing suspension concentrate to be obtained at 20% w/w solids. Similarly, azoxystrobin microparticles of approximately 2 μm diameter were also obtained using PGMA_50_-PTFEMA_80_ nanoparticles under the same conditions (see Figure S2).

**Figure 5:**
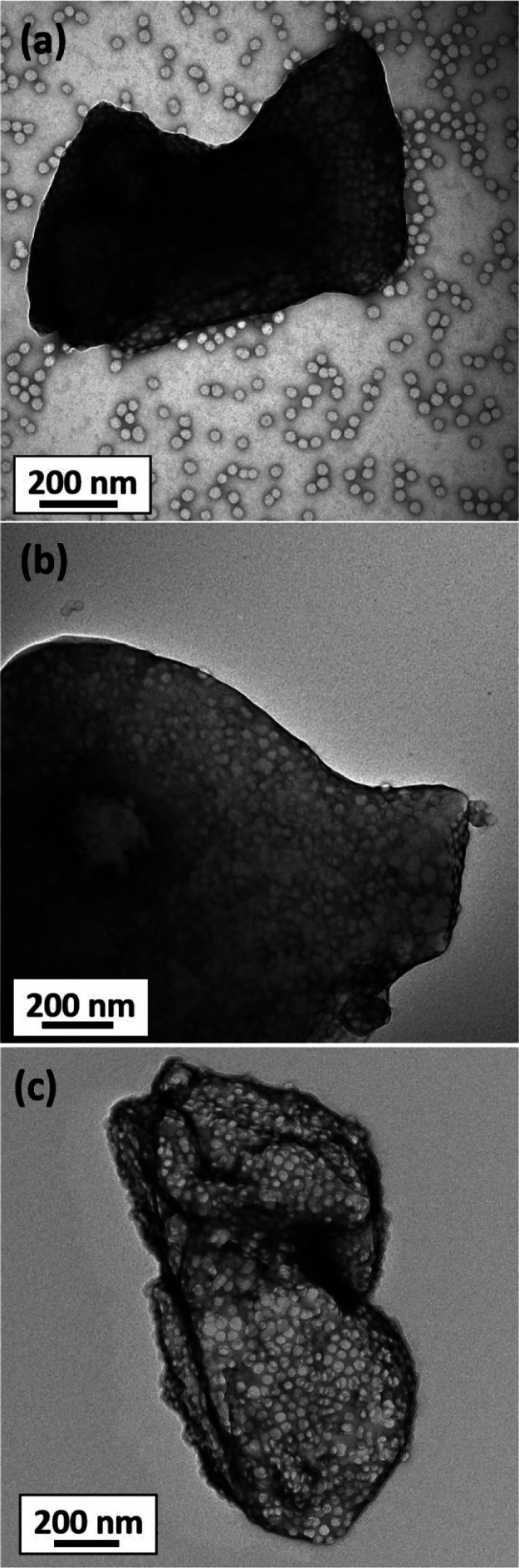
(a) TEM image recorded for azoxystrobin microparticles prepared by milling in the presence of PGMA_50_-PMMA_80_ nanoparticles before removal of excess non-adsorbed nanoparticles by centrifugation. (b) TEM image recorded for azoxystrobin microparticles prepared by milling in the presence of PGMA_50_-PMMA_80_ nanoparticles after removal of excess non-adsorbed nanoparticles by centrifugation. (c) TEM image recorded for azoxystrobin microparticles prepared by milling in the presence of PGMA_50_-PTFEMA_80_ nanoparticles after removal of excess non-adsorbed nanoparticles by centrifugation.

Figure 5a shows a TEM image recorded for the as-prepared azoxystrobin microparticles. The resulting PGMA_50_-PMMA_80_ nanoparticles were clearly present both on the crystal surface and also in the background. This suspension concentrate was then subjected to three centrifugation–redispersion cycles, and each supernatant was carefully decanted and discarded to remove any excess (non-adsorbed) nanoparticles. A TEM image recorded for the resulting purified azoxystrobin microparticles is shown in Figure 5b. Excess nanoparticles are no longer detected in the background, and the azoxystrobin microparticles are clearly coated with an adsorbed layer of PGMA_50_-PMMA_80_ nanoparticles. Similar observations were made for azoxystrobin microparticles milled in the presence of PGMA_50_-PTFEMA_80_ nanoparticles. Again, a relatively uniform layer of adsorbed nanoparticles is discernible at the surface of the azoxystrobin microparticles (Figure 5c).

**Figure 6:**
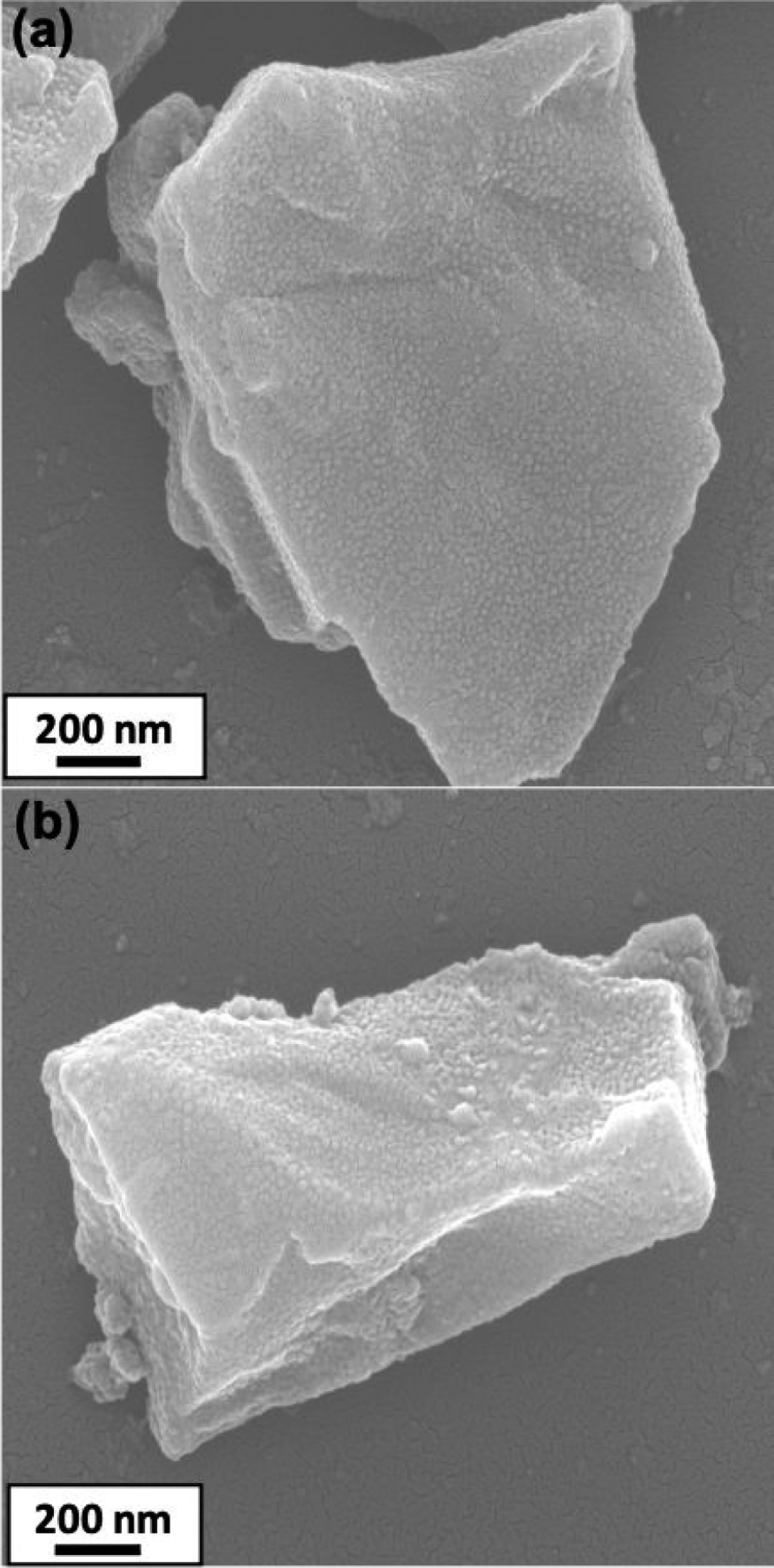
SEM images recorded for azoxystrobin microparticles coated with (a) PGMA_50_-PMMA_80_ nanoparticles and (b) PGMA_50_-PTFEMA_80_ nanoparticles. Both images were obtained after centrifugal purification to remove any excess non-adsorbed nanoparticles.

The nanoparticle-coated azoxystrobin microparticles were also characterized by scanning electron microscopy (see Figure 6a,b). A relatively uniform layer of adsorbed PGMA_50_-PMMA_80_ or PGMA_50_-PTFEMA_80_ nanoparticles (*z*-average diameter = 29 or 33 nm, respectively) is discernible at the surface of the micron-sized azoxystrobin crystals. Such SEM studies confirm that the nanoparticles survive the ball milling, regardless of the nature of the core-forming block.

**Figure 7:**
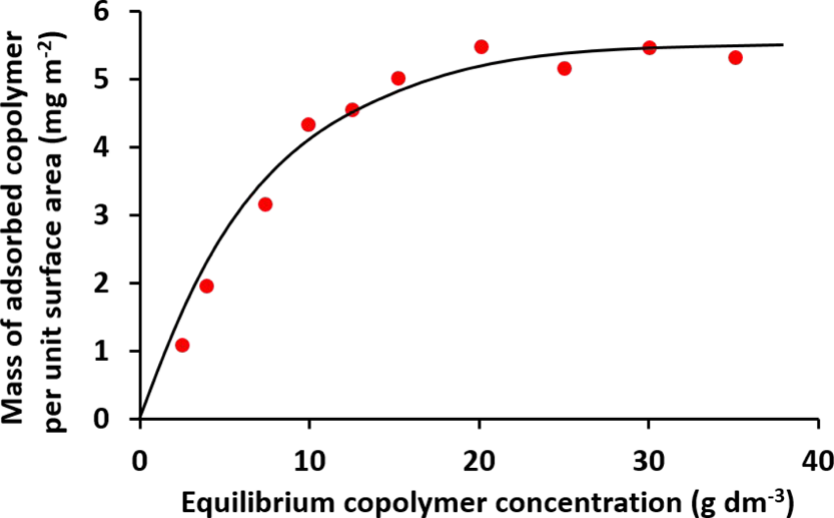
Langmuir-type adsorption isotherm constructed for PGMA_50_-PMMA_80_ nanoparticles adsorbed onto azoxystrobin microparticles at 20 °C as determined by a supernatant depletion assay based on solution densitometry.

The solution densities of aqueous dispersions of PGMA_50_-PMMA_80_ nanoparticles were determined at various concentrations using a solution densitometer to afford a linear calibration plot (see Figure S3). This enabled nanoparticle adsorption onto the azoxystrobin microparticles to be assessed indirectly using a supernatant depletion assay after sedimentation of the relatively large azoxystrobin microparticles by centrifugation, followed by analysis of the solution density of the remaining aqueous supernatant. Figure 7 shows the Langmuir-type adsorption isotherm constructed from such measurements. The maximum adsorbed amount, Γ, is around 5.5 mg m^–2^. A theoretical fractional surface coverage was calculated from this adsorbed amount using eq S1 (see the Supporting Information). This approach indicated a maximum fractional coverage of 0.25 for the PGMA_50_-PMMA_80_ nanoparticles. This value is comparable to that determined by Hayes and co-workers for the physical adsorption of 40 nm-diameter silica nanoparticles onto a planar aminated silicon wafer at pH 5.6 in the presence of 0.01 M KNO_3_ using optical reflectometry.^49^ A similar low-affinity-type isotherm (Γ = 3.8 mg m^–2^) was also obtained when using the PGMA_50_-PTFEMA_80_ nanoparticles under the same conditions (data not shown).

**Figure 8:**
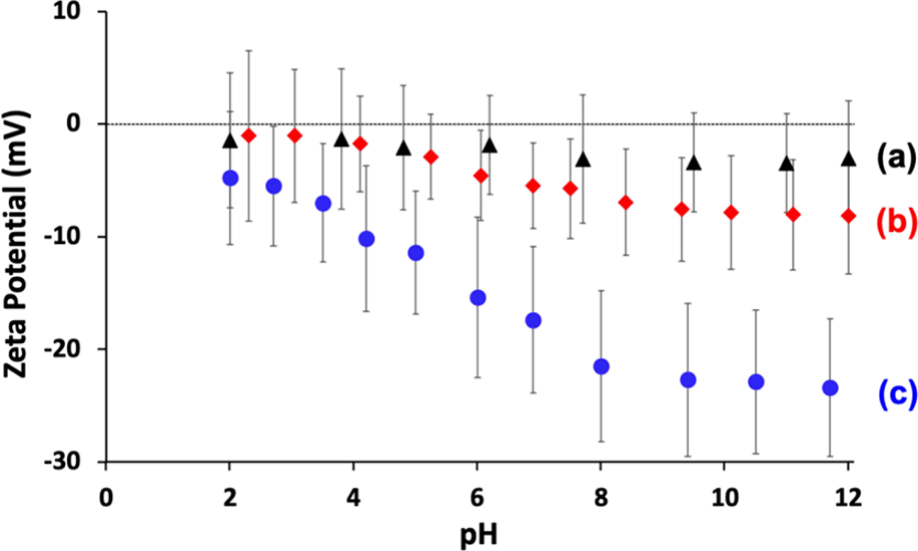
Zeta potential versus pH curves recorded for (a) aqueous dispersion of the PGMA_50_-PMMA_80_ nanoparticles alone, (b) diluted suspension concentrate comprising PGMA_50_-PMMA_80_ nanoparticle-coated azoxystrobin microparticles, and (c) coarse aqueous suspension comprising azoxystrobin crystals only. Thus, physical adsorption of the non-ionic PGMA_50_-PMMA_80_ nanoparticles significantly reduces the anionic surface character of azoxystrobin.

Zeta potential versus pH curves were determined for the PGMA_50_-PMMA_80_ nanoparticles, the nanoparticle-coated azoxystrobin microparticles, and the original azoxystrobin crystals (see Figure 8). The latter relatively coarse particles exhibited a zeta potential of around −23 mV above pH 9. In contrast, the PGMA_50_-PMMA_80_ nanoparticles exhibited zeta potentials close to zero (approximately −3 mV) across the whole pH range owing to the non-ionic nature of the PGMA steric stabilizer chains.^50^ Clearly, nanoparticle adsorption is not driven by electrostatics in the present study, which differentiates it from our earlier model system.^18^ Moreover, the significant reduction in the zeta potential (around −8 mV at pH 9–10) observed for the nanoparticle-coated anionic azoxystrobin microparticles provides further evidence for the partial surface coverage of the azoxystrobin microparticles by the near-neutral nanoparticles. Similar observations were made when using PGMA_50_-PTFEMA_80_ nanoparticles in place of the PGMA_50_-PMMA_80_ nanoparticles (Figure S4).

**Figure 9:**
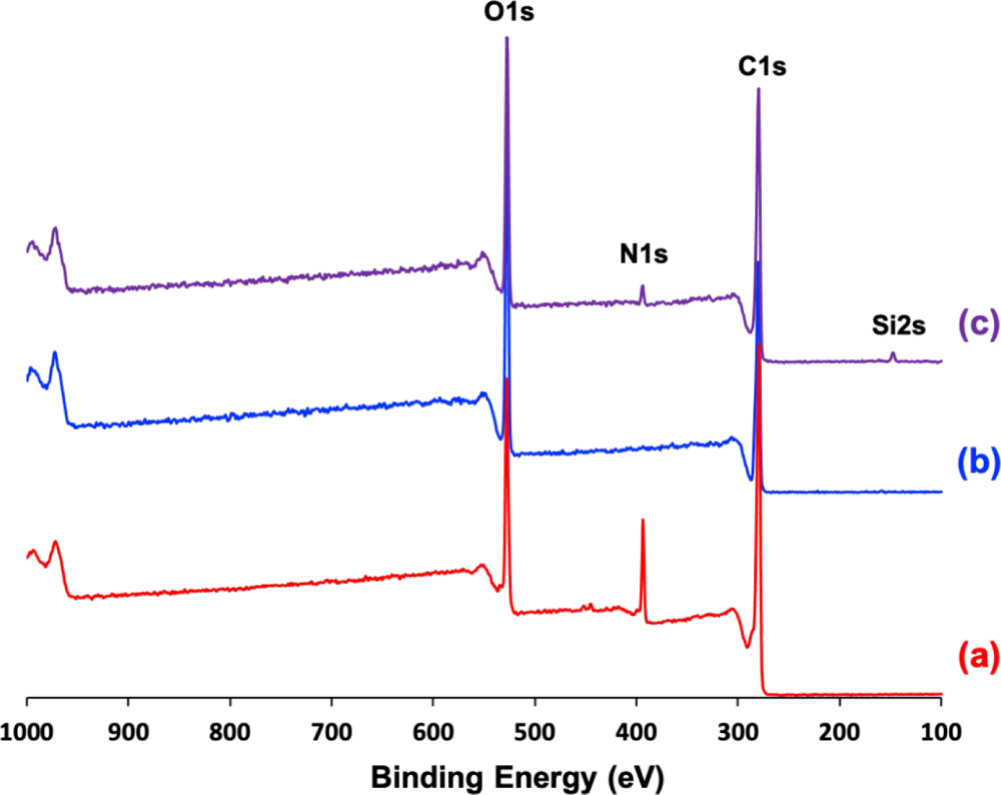
X-ray photoelectron survey spectra recorded for (a) pure azoxystrobin crystals, (b) PGMA_50_-PMMA_80_ nanoparticles alone, and (c) PGMA_50_-PMMA_80_ nanoparticle-coated azoxystrobin microparticles. These spectra confirm that the N1s signal may be used as a unique elemental marker for the azoxystrobin and that nanoparticle adsorption onto milled azoxystrobin microparticles leads to partial obscuration of this signal. Comparing the relative intensities of the N1s signals, the surface coverage of the azoxystrobin microparticles by the PGMA_50_-PMMA_80_ nanoparticles is estimated to be 0.24.

X-ray photoelectron survey spectra recorded for the azoxystrobin crystals, the PGMA_50_-PMMA_80_ nanoparticles alone, and the PGMA_50_-PMMA_80_ nanoparticle-coated azoxystrobin microparticles are shown in Figure 9. The chemical structure of azoxystrobin includes three nitrogen atoms (see Figure 1). In contrast, the PGMA_50_-PMMA_80_ nanoparticles contain no nitrogen atoms, so this element serves as a unique elemental marker for azoxystrobin (see Figure 9).^51^ If the azoxystrobin microparticles are partially coated with such nanoparticles and the mean nanoparticle diameter exceeds the X-ray photoelectron spectroscopy (XPS) sampling depth of 2–5 nm,^51^ then, the XPS N1s signal observed for the nanoparticle-coated azoxystrobin microparticles should be attenuated relative to that of azoxystrobin crystals alone. This is indeed the case: the former signal is 1.9 atom %, whereas the latter signal is 7.9 atom %. This implies a fractional surface coverage of approximately 1.9/7.9 = 0.24, which is consistent with the calculated theoretical surface coverage of 0.25 (see above). A comparable fractional surface coverage of 0.28 was calculated for the PGMA_50_-PTFEMA_80_ nanoparticle-coated azoxystrobin microparticles using the X-ray photoelectron survey spectra shown in Figure S5.

A control experiment was conducted whereby a suspension concentrate was prepared using a commercially available water-soluble polymer dispersant, Morwet D-425, rather than the nanoparticles described herein. Laser diffraction size distributions shown in Figure S6a confirm a similar reduction in the volume-average particle diameter to just under 2 μm for the azoxystrobin microparticles when using identical milling conditions. This mean size is consistent with images obtained by both optical microscopy and SEM (Figure S6b,c). Moreover, the latter technique indicates a smooth surface for the azoxystrobin microparticles, as expected when employing a soluble polymer as a comparable dispersant rather than nanoparticles. Clearly, sterically stabilized diblock copolymer nanoparticles offer dispersant performance to that achieved when using water-soluble polymers.

Finally, the nanoparticle-stabilized SCs reported herein were periodically sampled during storage at ambient temperature. Laser diffraction studies (data not shown) indicated no significant change in particle size over a six-month period, suggesting good long-term stability. On the other hand, addition of a non-ionic surfactant (Triton X-100) led to partial displacement of the adsorbed nanoparticles from the surface of the azoxystrobin nanoparticles (see Figure S7). Clearly, further studies are warranted to assess the long-term stability of such formulations under a range of conditions.

## Conclusions

Sterically stabilized diblock copolymer nanoparticles prepared by RAFT aqueous emulsion polymerization can be used as a dispersant for the preparation of micron-sized organic crystals via ball milling. This is exemplified for the specific case of azoxystrobin, a broad-spectrum fungicide that is widely used to prevent crop diseases. Electron microscopy studies confirm that the nanoparticles adsorb onto the azoxystrobin microparticles and modify their electrophoretic behavior. The extent of nanoparticle adsorption can be quantified using a supernatant assay based on solution densitometry. This indicates a maximum adsorbed amount of approximately 5.5 mg m^–2^, which suggests a theoretical surface coverage of 0.25. Moreover, XPS studies enable an experimental fractional surface coverage of approximately 0.24 as calculated from the attenuation of the underlying N1s signal arising from the azoxystrobin microparticles. Overall, this study suggests that sterically stabilized diblock copolymer nanoparticles may offer a useful alternative to traditional water-soluble copolymer dispersants in the formulation of SCs for agrochemical applications.

## Supporting Information Available

Schematic synthesis of PGMA_50_-PTFEMA_80_ nanoparticles; summary of GPC, DLS, and TEM data obtained for PGMA_50_-PTFEMA_80_ nanoparticles; laser diffraction curves recorded for azoxystrobin before and after milling in the presence of PGMA_50_-PTFEMA_80_ nanoparticles; linear calibration plot used for the solution densitometry studies; zeta potential versus pH curves recorded for PGMA_50_-PTFEMA_80_ nanoparticles, a diluted suspension concentrate comprising PGMA_50_-PTFEMA_80_ nanoparticle-coated azoxystrobin microparticles, and azoxystrobin crystals; X-ray photoelectron spectra recorded for pure azoxystrobin crystals, PGMA_50_-PTFEMA_80_ nanoparticles, and PGMA_50_-PTFEMA_80_ nanoparticle-coated azoxystrobin microparticles; summary of laser diffraction, optical microscopy, and SEM data obtained for azoxystrobin microparticles milled with Morwet D-425; TEM images recorded for azoxystrobin microparticles in the presence of nanoparticles after addition of the Triton X-100 surfactant; and equation used to calculate the theoretical fractional surface coverage of polymer nanoparticles (PDF)
