# Supplementary material for: Block Copolymer Nanoparticles are Effective Dispersants for Micrometer-Sized Organic Crystalline Particles
Source: ACS Appl Mater Interfaces. 2021 Jun 21;13(25):30235–43. doi: 10.1021/acsami.1c08261 (PMC8289232; doi:10.1021/acsami.1c08261)
Supplement: Supplementary file 1 — am1c08261_si_001.pdf [file am1c08261_si_001.pdf]

Supporting Information for:

Block Copolymer Nanoparticles are Effective Dispersants for Micrometer-sized Organic Crystalline Particles

Derek H. H. Chan,[a] Emily L. Kynaston,[b] Christopher Lindsay,[b] Philip Taylor[b] and Steven P. Armes[a]*

a. *Dainton Building, Department of Chemistry, University of Sheffield, Brook Hill, Sheffield, South Yorkshire, S3 7HF, UK.*

b. *Syngenta, Jealott's Hill International Research Centre, Bracknell, Berkshire, RG42 6EY, UK.*

Summary of Contents

Scheme S1. Synthesis of the $PGMA_{50}$-$PTFEMA_{80}$ nanoparticles used in this study.

Figure S1. GPC, DLS and TEM data obtained for the $PGMA_{50}$-$PTFEMA_{80}$ nanoparticles.

Figure S2. Laser diffraction particle size distribution curves obtained before and after milling of azoxystrobin with $PGMA_{50}$-$PTFEMA_{80}$ nanoparticles.

Figure S3. Calibration plot constructed for a series of aqueous dispersions of $PGMA_{50}$-$PMMA_{80}$ nanoparticles using solution densitometry.

Equation S1 and calculation of the theoretical surface coverage from the adsorbed amount.

Figure S4. Aqueous electrophoresis data recorded for azoxystrobin particles, $PGMA_{50}$-$PTFEMA_{80}$ nanoparticles and $PGMA_{50}$-$PTFEMA_{80}$ nanoparticle-coated azoxystrobin microparticles.

Figure S5. X-ray photoelectron survey spectra recorded for azoxystrobin particles, $PGMA_{50}$-$PTFEMA_{80}$ nanoparticles and $PGMA_{50}$-$PTFEMA_{80}$ nanoparticle-coated azoxystrobin microparticles.

Figure S6. Laser diffraction, optical microscopy image and SEM image obtained for azoxystrobin microparticles obtained using a commercial water-soluble polymer (Morwet D-425) as a dispersant.

Figure S7. TEM images recorded for azoxystrobin microparticles prepared using either $PGMA_{50}$-$PMMA_{80}$ or $PGMA_{50}$-$PTFEMA_{80}$ nanoparticles after addition of non-ionic Triton X-100 surfactant.

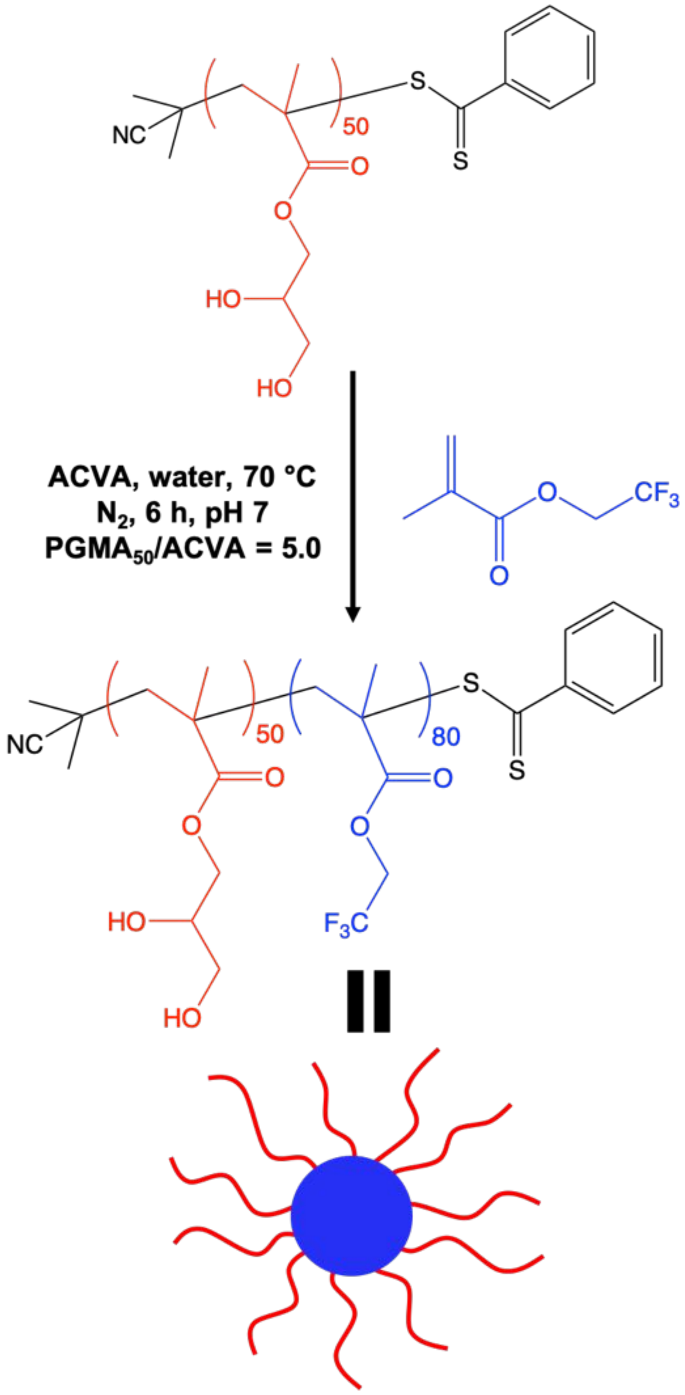

Scheme S1. Synthesis of $PGMA_{50}$-$PTFEMA_{80}$ diblock copolymer nanoparticles by RAFT aqueous emulsion polymerization of 2,2,2-trifluoroethyl methacrylate (TFEMA) at 70 °C.

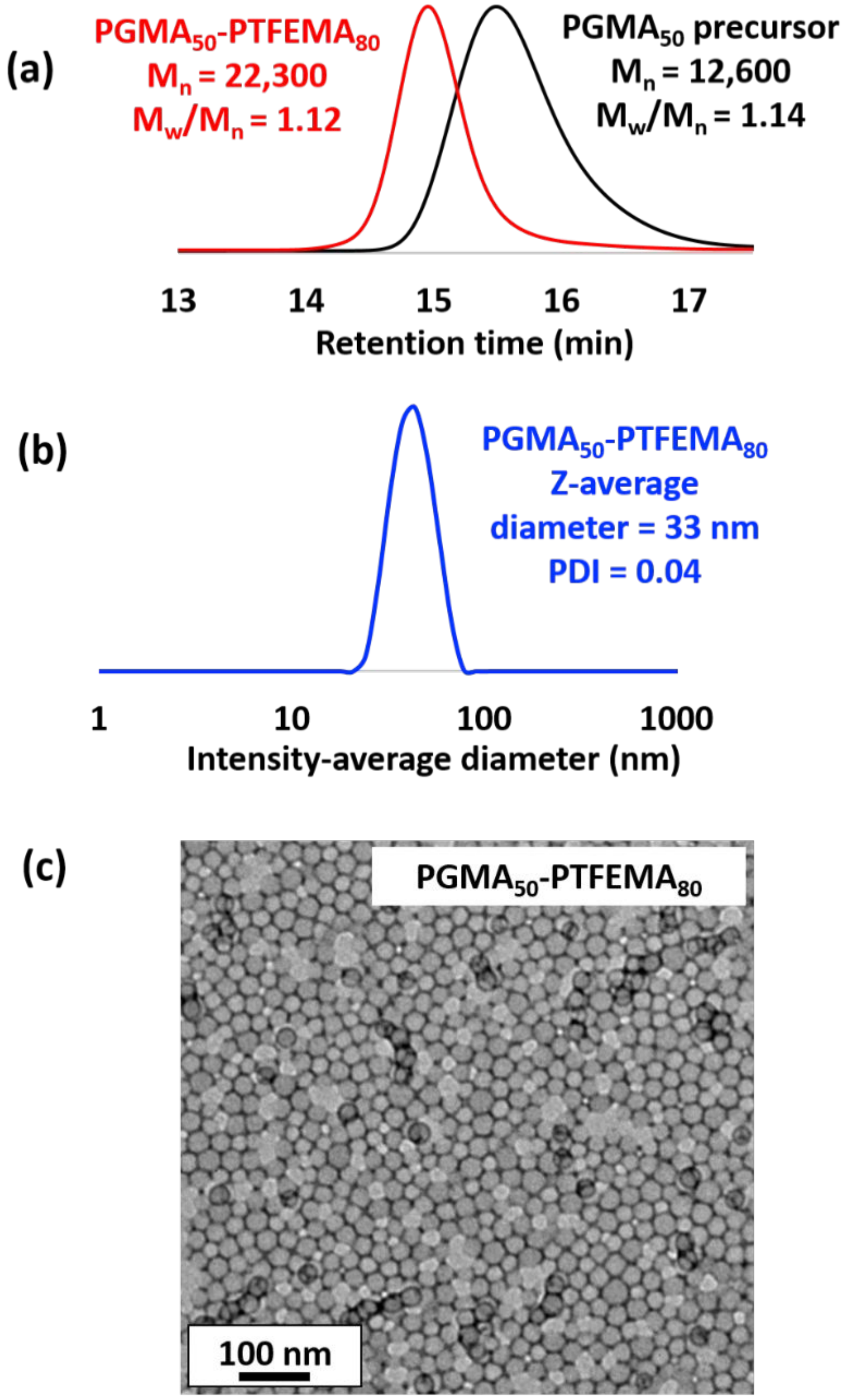

Fig. S1 (a) GPC curves recorded for the $PGMA_{50}$ precursor and $PGMA_{50}$-$PTFEMA_{80}$ nanoparticles; (b) DLS intensity-average particle size distribution (plus z-average diameter and polydispersity (PDI); (c) TEM image recorded for $PGMA_{50}$-$PTFEMA_{80}$ spherical nanoparticles.

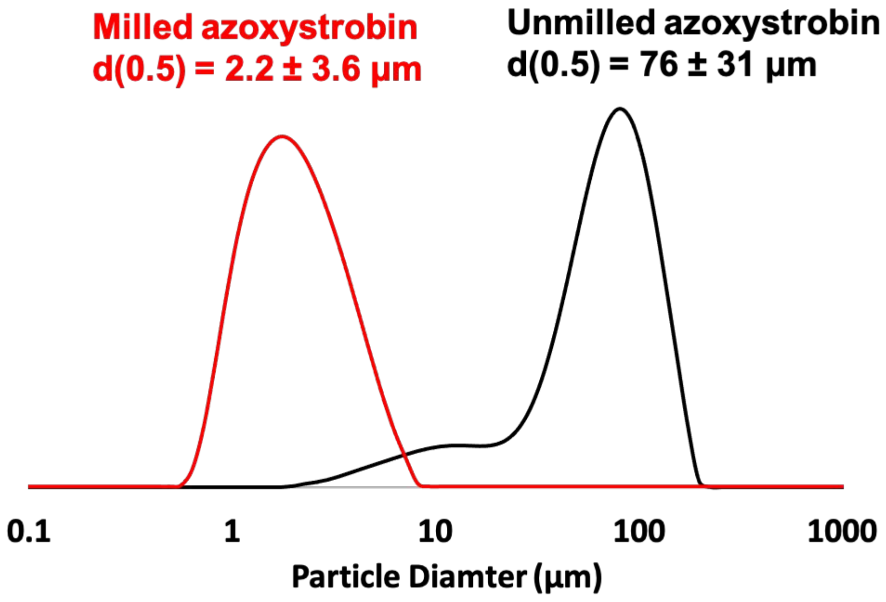

Fig. S2 Laser diffraction particle size distribution curves recorded for the original coarse azoxystrobin particles (black curve) and $PGMA_{50}$-$PTFEMA_{80}$ nanoparticle-coated azoxystrobin microparticles obtained after ball milling (red curve).

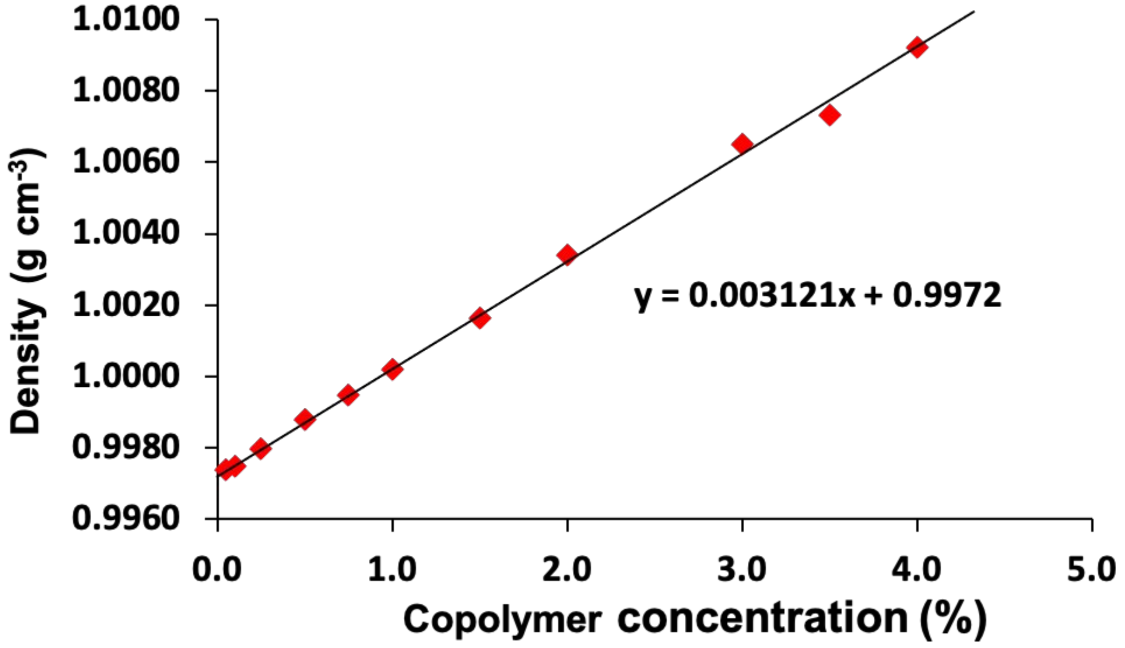

Fig. S3 Calibration plot constructed for solution density vs. $PGMA_{50}$-$PMMA_{80}$ nanoparticle concentration. This linear relationship was employed to calculate the concentration of nanoparticles remaining in the aqueous supernatant after milling various suspension concentrates followed by centrifugal sedimentation of the microparticles.

Calculation of the theoretical fractional surface coverage

The fractional surface coverage, θ, of the azoxystrobin microparticles by the nanoparticles was calculated using Equation **S1**:

$$\theta = \frac{3\Gamma}{4r\rho_p} \qquad \text{(S1)}$$

where Γ is the adsorbed amount of nanoparticles per unit area (g m^{-2}), r is the mean nanoparticle radius (m) and ρ_p is the nanoparticle density (g m^{-3}). For the $PGMA_{50}$-$PMMA_{80}$ nanoparticles, ρ_p was taken to be 1.1 x 10^6 g m^{-3} and r is estimated to be 1.5 x 10^{-8} m. If Γ is 5.5 mg m^{-2} (see Figure 7), then we calculate that the fractional surface coverage is 0.25.

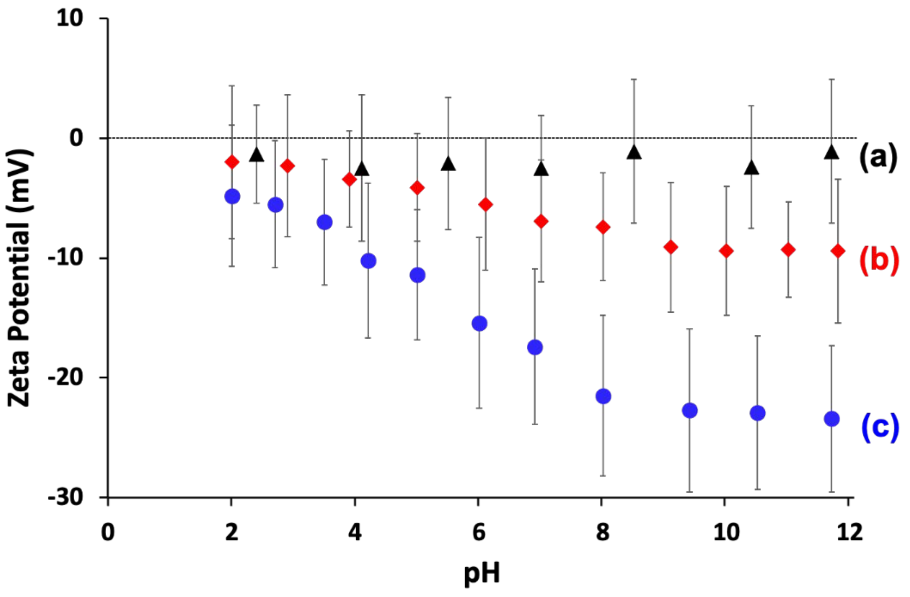

Fig. S4 Zeta potential vs. pH curves recorded for (a) an aqueous dispersion of the $PGMA_{50}$-$PTFEMA_{80}$ nanoparticles alone, (b) a diluted suspension concentrate comprising $PGMA_{50}$-$PTFEMA_{80}$ nanoparticle-coated azoxystrobin microparticles, and (c) a coarse aqueous suspension comprising azoxystrobin crystals only. Clearly, physical adsorption of the non-ionic $PGMA_{50}$-$PTFEMA_{80}$ nanoparticles significantly reduces the anionic surface character of the azoxystrobin.

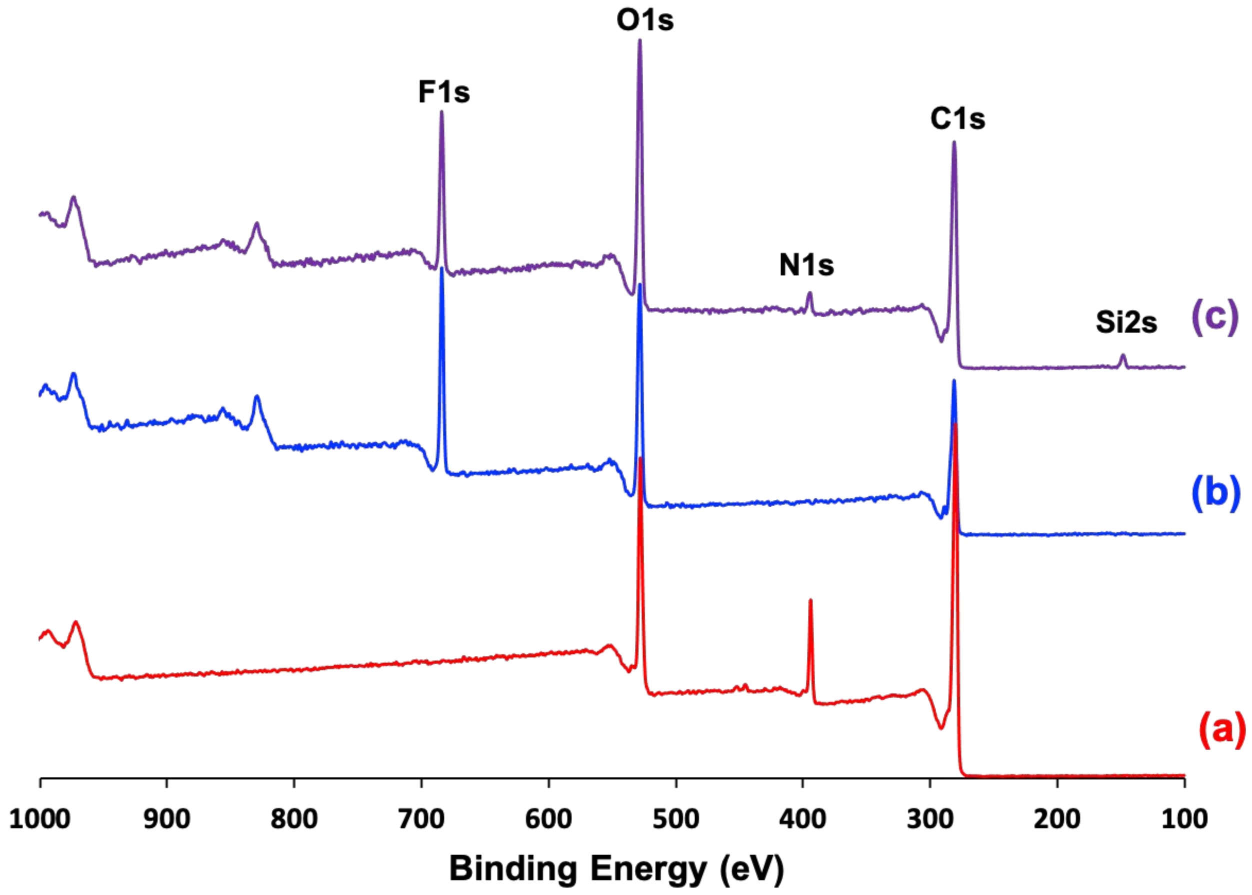

Fig. S5 X-ray photoelectron survey spectra recorded for (a) azoxystrobin crystals, (b) the $PGMA_{50}$-$PTFEMA_{80}$ nanoparticles alone and (c) the $PGMA_{50}$-$PTFEMA_{80}$ nanoparticle-coated azoxystrobin microparticles. These spectra confirm that the N1s signal can be used as a unique elemental marker for the azoxystrobin and that nanoparticle adsorption onto milled azoxystrobin microparticles leads to partial obscuration of this signal. Inspecting the relative intensities of the N1s signals, the surface coverage of the azoxystrobin microparticles by the $PGMA_{50}$-$PTFEMA_{80}$ nanoparticles is estimated to be 0.28.

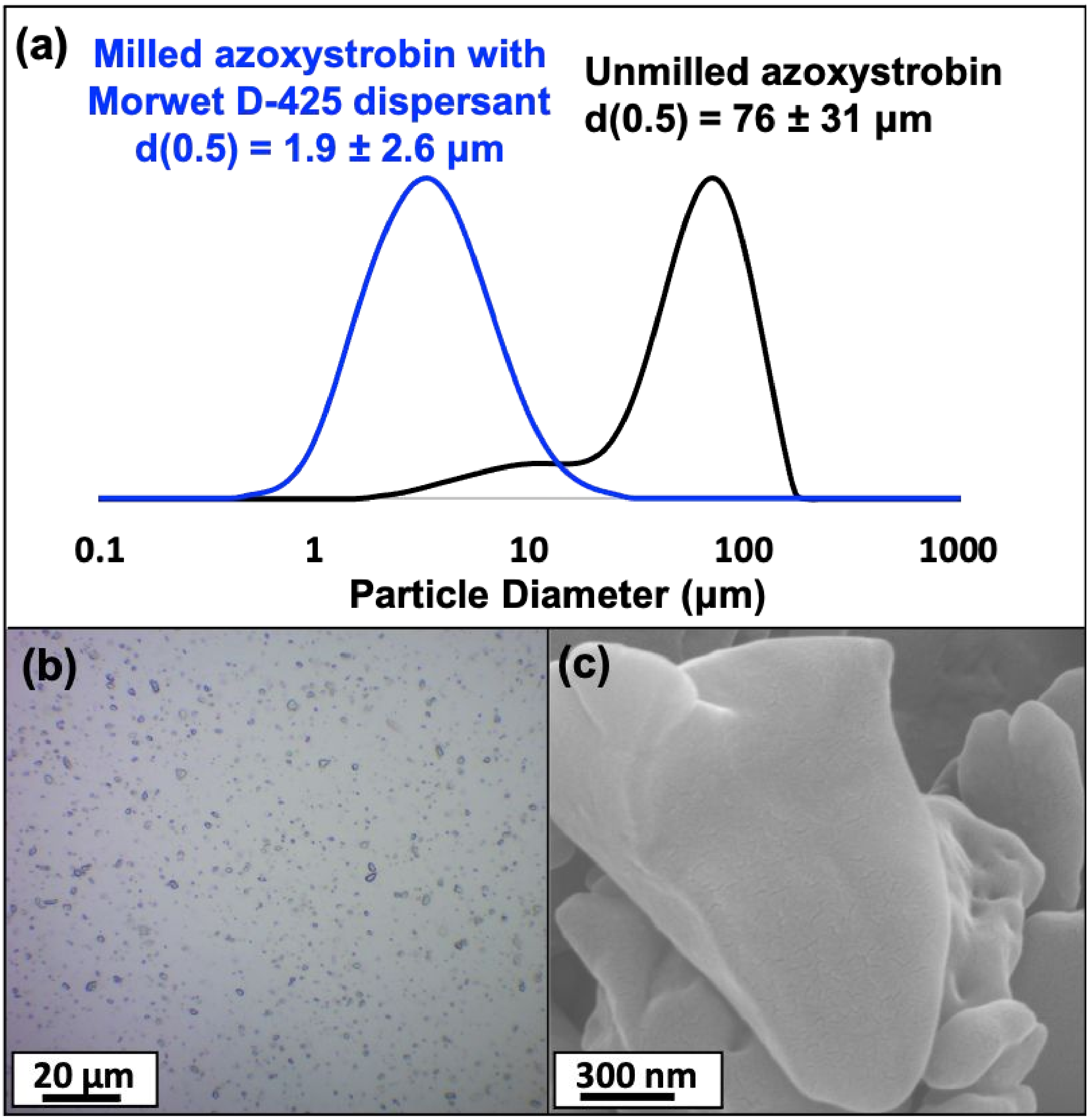

Fig. S6 (a) Laser diffraction particle size distributions recorded for the original coarse azoxystrobin particles and the much finer azoxystrobin microparticles obtained after ball milling in the presence of the commercial Morwet D-425 using the same conditions employed for the $PGMA_{50}$-$PMMA_{80}$ nanoparticles (see Figures 3 and 4). (b) Representative optical microscopy image and (c) SEM image recorded for azoxystrobin microparticles prepared using the Morwet D-425 dispersant. The latter technique confirms a smooth surface morphology, unlike that observed when using the nanoparticles as a particulate dispersant (see Figures 5 and 6).

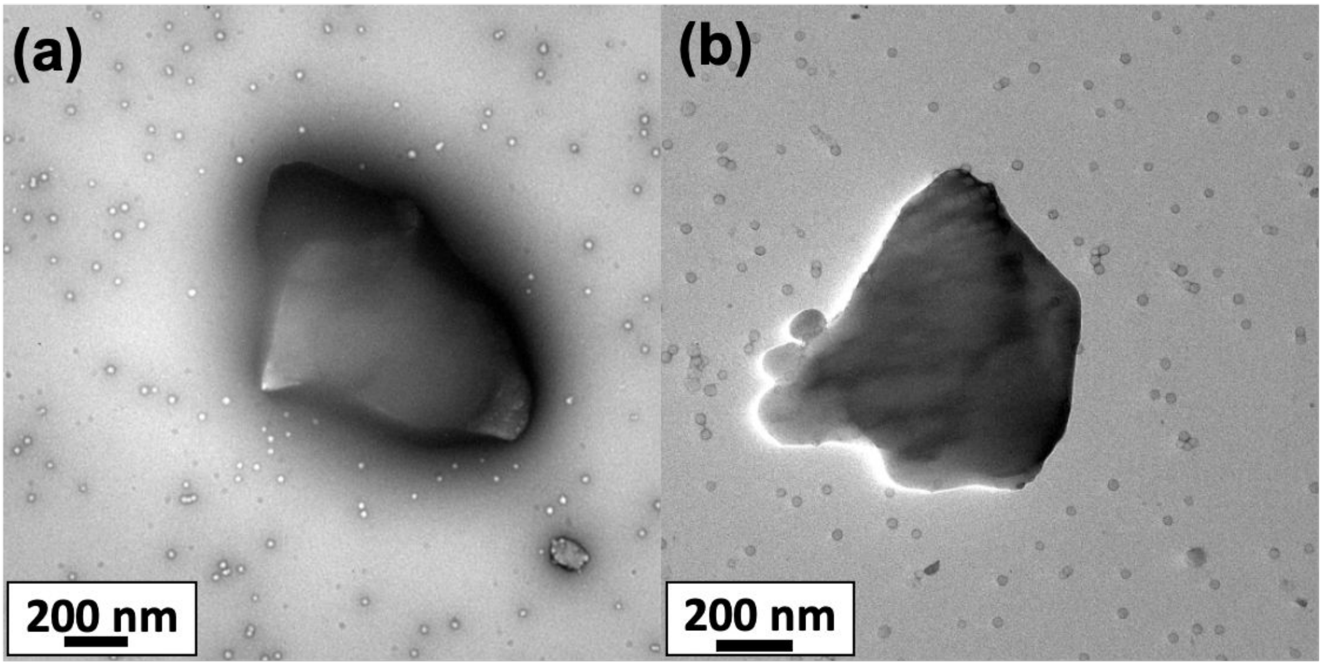

Fig. S7 TEM images recorded for azoxystrobin microparticles prepared using (a) $PGMA_{50}$-$PMMA_{80}$ and (b) $PTFEMA_{50}$-$PMMA_{80}$ nanoparticles after addition of Triton X-100 surfactant. In each case, the presence of this non-ionic surfactant clearly leads to (partial) displacement of the adsorbed nanoparticles from the surface of the azoxystrobin microparticles.
